# Relative Survival After Transcatheter Aortic Valve Implantation: How Do Patients Undergoing Transcatheter Aortic Valve Implantation Fare Relative to the General Population?

**DOI:** 10.1161/JAHA.117.007229

**Published:** 2017-10-17

**Authors:** Glen P. Martin, Matthew Sperrin, William Hulme, Peter F. Ludman, Mark A. de Belder, William D. Toff, Oras Alabas, Neil E. Moat, Sagar N. Doshi, Iain Buchan, John E. Deanfield, Chris P. Gale, Mamas A. Mamas, the National Institute of Cardiovascular Outcomes Research (NICOR)

**Affiliations:** ^1^ Farr Institute Faculty of Biology, Medicine and Health Manchester Academic Health Science Centre University of Manchester United Kingdom; ^2^ Queen Elizabeth Hospital Birmingham United Kingdom; ^3^ James Cook University Hospital Middlesbrough United Kingdom; ^4^ Department of Cardiovascular Sciences Clinical Sciences Wing Glenfield General Hospital University of Leicester United Kingdom; ^5^ National Institute for Health Research (NIHR) Leicester Cardiovascular Biomedical Research Unit Leicester United Kingdom; ^6^ Leeds Institute of Cardiovascular and Metabolic Medicine University of Leeds United Kingdom; ^7^ Royal Brompton and Harefield National Health Service Foundation Trust London United Kingdom; ^8^ National Institute for Cardiovascular Outcomes Research University College London London United Kingdom; ^9^ Keele Cardiovascular Research Group Institute of Applied Clinical Science and Centre for Prognosis Research Keele University Stoke‐on‐Trent United Kingdom

**Keywords:** aortic stenosis, mortality, relative survival, transcatheter aortic valve implantation, Mortality/Survival, Aortic Valve Replacement/Transcather Aortic Valve Implantation

## Abstract

**Background:**

Transcatheter aortic valve implantation (TAVI) is indicated for patients with aortic stenosis who are intermediate‐high surgical risk. Although all‐cause mortality rates after TAVI are established, survival attributable to the procedure is unclear because of competing causes of mortality. The aim was to report relative survival (RS) after TAVI, which accounts for background mortality risks in a matched general population.

**Methods and Results:**

National cohort data (n=6420) from the 2007 to 2014 UK TAVI registry were matched by age, sex, and year to mortality rates for England and Wales (population, 57.9 million). The Ederer II method related observed patient survival to that expected from the matched general population. We modelled RS using a flexible parametric approach that modelled the log cumulative hazard using restricted cubic splines. RS of the TAVI cohort was 95.4%, 90.2%, and 83.8% at 30 days, 1 year, and 3 years, respectively. By 1‐year follow‐up, mortality hazards in the >85 years age group were not significantly different from those of the matched general population; by 3 years, survival rates were comparable. The flexible parametric RS model indicated that increasing age was associated with significantly lower excess hazards after the procedure; for example, by 2 years, a 5‐year increase in age was associated with 20% lower excess mortality over the general population.

**Conclusions:**

RS after TAVI was high, and survival rates in those aged >85 years approximated those of a matched general population within 3 years. High rates of RS indicate that patients selected for TAVI tolerate the risks of the procedure well.

**Clinical Perspective**

**What Is New?**

- Relative survival (RS) adjusts observed mortality, for mortality rates expected within an age‐, sex‐, year‐ and region‐matched general population.
- This is the first study to apply RS in transcatheter aortic valve implantation (TAVI), and demonstrates good long‐term RS rates in patients selected for the procedure.
- RS was particularly high in elderly patients, in whom observed mortality hazards returned to that seen in a matched general population by 1 year after TAVI.
- High RS within elderly patients with TAVI indicates that a large proportion of long‐term deaths can be accounted for by the underlying mortality risks within the general population.

**What Are the Clinical Implications?**

- Elderly patients with severe aortic stenosis, who are selected to undergo TAVI, tolerate the risks of the procedure well, which has important implications from a resource allocation perspective.
- The careful selection applied to elderly TAVI candidates appears to control procedure‐related mortality rates, meaning that elderly patients should continue to be considered for TAVI, even with the potential expansion into lower‐risk patients.
- Future reporting of mortality rates from national TAVI registries should be presented in the context of the patient population, particularly when comparing rates across countries where underlying expected mortality may vary considerably.

## Introduction

Aortic stenosis (AS) is the most common valve pathological feature in Europe and North America and occurs because of age‐related degeneration and calcification of the aortic valve. The onset of AS symptoms is associated with poor prognosis, with an estimated annual mortality rate of 25%.^1^ Although surgical valve replacement is the mainstay treatment for AS, randomized controlled trials have demonstrated the efficacy of transcatheter aortic valve implantation (TAVI) for symptomatic patients with AS considered to be at intermediate to high operative risk.^2^, ^3^, ^4^, ^5^ Similarly, studies on several national TAVI registries have shown favorable short‐ and mid‐term mortality.^6^, ^7^, ^8^, ^9^ However, because TAVI is recommended in those at high risk, the patients who undergo such a procedure are older and have more comorbid conditions than those undergoing alternative treatment options. Consequently, the long‐term mortality profile is difficult to assess in patients undergoing TAVI because, by virtue of age and multimorbidity, their risk of dying from other causes is high.

Administrative data on cause of death could be used to estimate the mortality profile associated with the disease or treatment in question, but such data are often unreliable.^10^ An alternative method is relative survival (RS), which adjusts the observed mortality for the expected mortality rates within a matched general population.^11^, ^12^ Specifically, overall excess mortality attributable to the index AS and associated TAVI can be estimated as the difference between observed and expected mortality, which forms the RS estimate. Although RS is commonly used in studies of survival after cancer diagnosis^13^ and is beginning to be used in cardiovascular disease,^14^, ^15^ to date such methods have not been used to assess long‐term mortality outcomes after TAVI.

The aim of this study was to investigate the survival of patients treated by TAVI in a national cohort, while adjusting for underlying expected mortality risks within a matched general population.

## Methods

### UK TAVI Registry

Prospective data on all consecutive TAVI procedures in the United Kingdom are collected in the UK registry through a Web‐based interface provided by the National Institute of Cardiovascular Outcomes Research; individual TAVI centers are contacted if there are data inconsistencies. Further details of the registry have been published previously.^16^ In summary, 95 variables are recorded, detailing patient demographics, risk factors for intervention, procedural details, and adverse outcomes up to the time of hospital discharge. This study included all consecutive TAVI procedures conducted between January 1, 2007 and December 31, 2014 across the 32 centers running active TAVI programs in England and Wales.

All‐cause mortality was obtained from the Office for National Statistics, providing the life status of English and Welsh patients. Although mortality information was available in most cases, we excluded patients with missing life status. Administrative censoring occurred at the end of follow‐up on May 31, 2015. All survival times were defined as the number of days between the date of TAVI and either the date of death or the date of last information on follow‐up, up to a maximum of 3 years after procedure.

### Statistical Analysis

RS was defined as the observed survival from the TAVI registry divided by the expected survival from a matched general population. RS equal to 1 indicates that the observed survival is the same as that within the matched general population, whereas RS <1 means that the observed survival is worse than that expected within the general population. This study used the population life tables provided by the Office for National Statistics to derive the population‐expected survival; such life tables are stratified by age, sex, year, and country (ie, different tables for England and Wales). Although the life table mortality estimates will include those patients undergoing TAVI, the low prevalence of such procedures undertaken across the general population means that the bias induced by this will be negligible.^11^ Furthermore, although the UK TAVI registry had time‐to‐event information censored in 2015, the life tables were only available to 2014. Hence, the expected population mortality rates for 2015 were assumed the same as those from 2014. We excluded patients who were missing data on age, sex, year, or country, which were used to match to the life tables. All analyses were conducted in the whole TAVI cohort and across the following subgroups: age (<80, 80–85, and >85 years), procedure year (2007–2010 and 2011–2014), and sex.

The Ederer II method was used to estimate RS,^11^ from which an estimate of the cumulative excess hazard could be obtained. An increasing (decreasing) cumulative excess hazard through time indicates that the mortality hazard in the TAVI population was higher (lower) than that expected from the matched general population. Constant cumulative excess hazards infer that the observed mortality hazard was the same as that expected from the general population (ie, observed hazards of mortality “returned to baseline”); a cumulative excess hazard of 0 means that observed survival equals the expected survival (ie, RS=1).

In addition, we calculated expected daily mortality hazards using the life tables and observed daily mortality hazards using the Kaplan‐Meier estimate. An estimated daily hazard ratio (HR) was then obtained by dividing the observed and expected daily hazard rates at each follow‐up time within the first year of the procedure. Herein, a daily HR of 1 implies the observed mortality hazards were the same as those in the general population, whereas HRs <1 (>1) imply that mortality hazards after TAVI were lower (higher) than the general population hazard. A smoother was applied to each of the daily HRs to estimate a trend through time.^17^

An alternative estimation of RS was considered that uses a patient‐level approach, which has previously been described in detail.^18^ In short, the follow‐up time (in days) for each patient was transformed to give the expected proportion of the matched general population that would have not survived that individual's follow‐up. For example, transforming the observed survival time for a given patient to 0.3 implies that the individual survived >30% of their matched general population. A Kaplan‐Meier estimate and log‐rank test can then be used directly on the transformed survival times to compare across strata.

Multivariable modelling of RS was undertaken using a flexible parametric RS model, which modelled the log cumulative hazard using restricted cubic splines, thereby allowing the baseline hazard to vary nonlinearly with time.^19^, ^20^ Covariates in the model included age (continuous variable), procedure year (continuous variable), and sex. Other demographic and procedural variables were not entered into the model because our aim was not to identify a causal relationship of age, year, or sex on RS; rather, the aim was to explore associations of such variables. Interaction terms between log survival time and age, year, and sex were included, to estimate time‐varying excess HRs. The degrees of freedom for the baseline and time‐varying coefficients were selected to minimize the Akaike Information Criterion, with the knots of the splines placed at the centiles of the uncensored survival times.

R version 3.3.1^21^ was used for all statistical analyses. Graphical plots were made using the “ggplot2” package,^22^ the package “rstpm2” was used to fit the flexible parametric RS models,^23^ and the package “relsurv” was used for the Ederer II RS analysis.^24^

## Results

Between January 1, 2007 and December 31, 2014, 6835 patients received TAVI in either England or Wales; patients with missing location information (n=337) were removed from the analysis. Of the remaining 6498 patients, 56 had missing follow‐up time and 22 had missing sex information, leaving 6420 patients (94%) available for analysis. Tables 1, 2 through 3 present baseline characteristics of the whole TAVI cohort and across age, procedure year and sex subgroups, respectively. The mean age of patients was 81.3 years, and most patients were male (53.7%). Older patients generally had fewer baseline risk factors (Table 1); for instance, the prevalence of renal failure (*P*<0.001), asthma/chronic obstructive pulmonary disease (*P*<0.001), extracardiac arteriopathy (*P*<0.001), and left ventricular ejection fraction <50% (*P*<0.001) was significantly lower for older patients. The median follow‐up was 710 days (interquartile range, 351–1219 days), with 14 627 person‐years of follow‐up.

**Table 1:** Baseline and Procedural Characteristics of the UK TAVI Data Set and Across Age Subgroups

Variable	TAVI Cohort (n=6420)	Aged <80 y (n=2213)	Aged 80–85 y (n=1754)	Aged ≥85 y (n=2453)	*P* Value^a^
Age, mean (range), y	81.3 (29–101)	73.1 (29–79)	82.2 (80–84)	88.0 (85–101)	<0.001
Female sex, n (%)	2972 (46.3)	867 (39.2)	788 (44.9)	1317 (53.7)	<0.001
Diabetes mellitus, n (%)					<0.001
Nondiabetic	4908 (76.4)	1520 (68.7)	1323 (75.4)	2065 (84.2)	
Dietary control	282 (4.39)	105 (4.74)	78 (4.45)	99 (4.04)	
Oral medicine	844 (13.1)	379 (17.1)	250 (14.3)	215 (8.76)	
Insulin	351 (5.47)	198 (8.95)	91 (5.19)	62 (2.53)	
Current or ex‐smoker, n (%)	3313 (51.6)	1306 (59.0)	923 (52.6)	1084 (44.2)	<0.001
Creatinine, mean (range), μmol/L	114.3 (29.0–1044.0)	118.8 (39–1044)	115.4 (38–681)	109.4 (29–554)	<0.001
Renal failure^b^	390 (6.07)	169 (7.64)	118 (6.73)	103 (4.20)	<0.001
MI, n (%)					
Within 90 d of TAVI	145 (2.26)	51 (2.30)	39 (2.22)	55 (2.24)	0.984
Within 30 d of TAVI	62 (0.97)	24 (1.08)	18 (1.03)	20 (0.82)	0.607
Asthma/COPD, n (%)	1679 (26.2)	744 (33.6)	472 (26.9)	463 (18.9)	<0.001
Extracardiac arteriopathy, n (%)	1519 (23.7)	603 (27.2)	422 (24.1)	494 (20.1)	<0.001
Calcification of ascending aorta, n (%)	1173 (18.3)	428 (19.3)	345 (19.7)	400 (16.3)	0.005
Atrial fibrillation/flutter, n (%)	1568 (24.4)	482 (21.8)	466 (26.6)	620 (25.3)	<0.001
Previous cardiac surgery, n (%)	1999 (31.1)	1009 (45.6)	611 (34.8)	379 (15.5)	<0.001
Previous PCI, n (%)	1300 (20.2)	475 (21.5)	371 (21.2)	454 (18.5)	0.020
Weight, mean (range), kg	74.0 (32.0–190.0)	80.1 (32–190)	74.0 (33–141.7)	68.5 (32–163)	<0.001
Height, mean (range), m	1.65 (1.10–2.36)	1.67 (1.10–2.01)	1.65 (1.15–1.97)	1.63 (1.16–2.36)	<0.001
Critical preoperative state, n (%)	105 (1.64)	50 (2.26)	25 (1.43)	30 (1.22)	0.015
CCS class 4, n (%)	77 (1.20)	30 (1.36)	19 (1.08)	28 (1.14)	0.698
NYHA class ≥III	5140 (80.1)	1776 (80.3)	1413 (80.6)	1951 (79.5)	0.593
LVEF, n (%)					<0.001
≥50%	3907 (60.9)	1246 (56.3)	1066 (60.8)	1595 (65.0)	
30%–49%	1870 (29.1)	676 (30.5)	502 (28.6)	692 (28.2)	
*<*30%	585 (9.11)	272 (12.3)	165 (9.41)	148 (6.03)	
Procedure urgency, n (%)					0.161
Elective	5624 (87.6)	1911 (86.4)	1550 (88.4)	2163 (88.2)	
Urgent	749 (11.7)	280 (12.7)	193 (11.0)	276 (11.3)	
Emergency/salvage, n (%)	40 (0.62)	19 (0.86)	8 (0.46)	13 (0.53)	
Valve type, n (%)					0.248
Edwards SAPIEN	3496 (54.5)	1179 (53.3)	938 (53.5)	1379 (56.2)	
Medtronic CoreValve	2680 (41.7)	948 (42.8)	745 (42.5)	987 (40.2)	
Other	215 (3.35)	75 (3.39)	64 (3.65)	76 (3.10)	
Access route, n (%)					<0.001
TF access	4795 (74.7)	1583 (71.5)	1309 (74.6)	1903 (77.6)	
TA access	1009 (15.7)	402 (18.2)	279 (15.9)	328 (13.4)	
Other access	604 (9.41)	225 (10.2)	161 (9.18)	218 (8.89)	
Logistic EuroSCORE, mean (range)^c^	21.9 (1.51–93.6)	18.4 (1.51–86.0)	23.2 (5.83–91.1)	24.0 (7.96–93.6)	<0.001
STS score, mean (range)^c^	5.06 (0.46–55.4)	3.78 (0.46–44.6)	5.04 (1.15–55.4)	6.22 (1.49–44.8)	<0.001

CCS indicates coronary calcification score; COPD, chronic obstructive pulmonary disease; LVEF, left ventricular ejection fraction; MI, myocardial infarction; NYHA, New York Heart Association Functional Classification; PCI, percutaneous coronary intervention; STS, Society of Thoracic Surgeons; TA, transapical; TAVI, transcatheter aortic valve implantation; and TF, transfemoral.

a *P* value indicates the age group comparison.

b Defined as creatinine >200 μmol/L or dialysis for renal failure.

c The logistic EuroSCORE and the STS score predict the risk of 30‐day mortality using a range of risk factors known before the operation.

**Table 2:** Baseline Characteristics Across Procedure Year Subgroups

Variable	Procedure Year 2007–2010 (n=1528)	Procedure Year 2011–2014 (n=4892)	*P* Value
Age, mean (range), y	81.5 (44–99)	81.2 (29–101)	0.217
Female sex, n (%)	721 (47.2)	2251 (46.0)	0.440
Diabetes mellitus, n (%)			0.184
Nondiabetic	1188 (77.7)	3720 (76.0)	
Dietary control	74 (4.84)	208 (4.25)	
Oral medicine	178 (11.6)	666 (13.6)	
Insulin	86 (5.63)	265 (5.42)	
Current or ex‐smoker, n (%)	846 (55.4)	2467 (50.4)	0.021
Creatinine, mean (range), μmol/L	118.5 (37–736)	113.0 (29–1044)	0.004
Renal failure^a^, n (%)*	109 (7.13)	281 (5.74)	0.073
MI, n (%)			
Within 90 d of TAVI	39 (2.55)	106 (2.17)	0.452
Within 30 d of TAVI	18 (1.18)	44 (0.90)	0.424
Asthma/COPD, n (%)	421 (27.6)	1258 (25.7)	0.112
Extracardiac arteriopathy, n (%)	418 (27.4)	1101 (22.5)	<0.001
Calcification of ascending aorta, n (%)	372 (24.3)	801 (16.4)	<0.001
Atrial fibrillation/flutter, n (%)	359 (23.5)	1209 (24.7)	0.338
Previous cardiac surgery, n (%)	483 (31.6)	1516 (31.0)	0.795
Previous PCI, n (%)	332 (21.7)	968 (19.8)	0.136
Weight, mean (range), kg	72.2 (33–153)	74.6 (32–190)	<0.001
Height, mean (range), m	1.64 (1.10–1.90)	1.64 (1.14–2.36)	0.054
Critical preoperative state, n (%)	25 (1.64)	80 (1.64)	0.999
CCS class 4, n (%)	26 (1.70)	51 (1.04)	0.057
NYHA class ≥III	1249 (81.7)	3891 (79.5)	0.161
LVEF, n (%)			0.322
≥50%	954 (62.4)	2953 (60.4)	
30%–49%	423 (27.7)	1447 (29.6)	
*<*30%	138 (9.03)	447 (9.14)	
Procedure urgency, n (%)			<0.001
Elective	1385 (90.6)	4239 (86.7)	
Urgent	136 (8.90)	613 (12.5)	
Emergency/salvage	7 (0.46)	33 (0.67)	
Valve type, n (%)			<0.001
Edwards SAPIEN	760 (49.7)	2736 (55.9)	
Medtronic CoreValve	762 (49.9)	1918 (39.2)	
Other	0 (0)	215 (4.39)	
Access route, n (%)			<0.001
TF access	1029 (67.3)	3766 (77.0)	
TA access	389 (25.5)	620 (12.7)	
Other access	110 (7.20)	494 (10.1)	

CCS indicates coronary calcification score; COPD, chronic obstructive pulmonary disease; LVEF, left ventricular ejection fraction; MI, myocardial infarction; NYHA, New York Heart Association Functional Classification; PCI, percutaneous coronary intervention; TA, transapical; TAVI, transcatheter aortic valve implantation; and TF, transfemoral.

a Defined as creatinine >200 μmol/L or dialysis for renal failure.

**Table 3:** Baseline Characteristics Across Sex Subgroups

Variable	Women (n=2972)	Men (n=3448)	*P* Value
Age, mean (range), y	82.3 (30–100)	80.4 (29–101)	<0.001
Diabetes mellitus, n (%)			<0.001
Nondiabetic	2358 (79.3)	2550 (74.0)	
Dietary control	119 (4.00)	163 (4.73)	
Oral medicine	338 (11.4)	506 (14.7)	
Insulin	142 (4.78)	209 (6.06)	
Current or ex‐smoker, n (%)	1095 (36.8)	2218 (64.3)	<0.001
Creatinine, mean (range), μmol/L	100.2 (29–649)	126.4 (39–1044)	<0.001
Renal failure^a^, n (%)*	109 (3.67)	281 (8.15)	<0.001
MI, n (%)			
Within 90 d of TAVI	60 (2.02)	85 (2.47)	0.262
Within 30 d of TAVI	22 (0.74)	40 (1.16)	0.112
Asthma/COPD, n (%)	743 (25.0)	936 (27.1)	0.044
Extracardiac arteriopathy, n (%)	560 (18.8)	959 (27.8)	<0.001
Calcification of ascending aorta, n (%)	578 (19.4)	595 (17.3)	0.029
Atrial fibrillation/flutter, n (%)	676 (22.7)	892 (25.9)	0.003
Previous cardiac surgery, n (%)	521 (17.5)	1478 (42.9)	<0.001
Previous PCI, n (%)	487 (16.4)	813 (23.6)	<0.001
Weight, mean (range), kg	67.5 (32–153)	79.6 (38–190)	<0.001
Height, mean (range), m	1.57 (1.10–1.94)	1.71 (1.15–2.36)	<0.001
Critical preoperative state, n (%)	43 (1.45)	62 (1.80)	0.307
CCS class 4, n (%)	35 (1.18)	42 (1.22)	0.972
NYHA class ≥III	2413 (81.2)	2727 (79.1)	0.041
LVEF, n (%)			<0.001
≥50%	2067 (69.5)	1840 (53.4)	
30%–49%	731 (24.6)	1139 (33.0)	
<30%	147 (4.95)	438 (12.7)	
Procedure urgency, n (%)			0.460
Elective	2613 (87.9)	3011 (87.3)	
Urgent	340 (11.4)	409 (11.9)	
Emergency/salvage	15 (0.50)	25 (0.73)	
Valve type, n (%)			0.546
Edwards SAPIEN	1607 (54.1)	1889 (54.8)	
Medtronic CoreValve	1244 (41.9)	1436 (41.6)	
Other	107 (3.60)	108 (3.13)	
Access route, n (%)			0.013
TF access	2270 (76.4)	2525 (73.2)	
TA access	430 (14.5)	579 (16.8)	
Other access	267 (8.98)	337 (9.77)	

CCS indicates coronary calcification score; COPD, chronic obstructive pulmonary disease; LVEF, left ventricular ejection fraction; MI, myocardial infarction; NYHA, New York Heart Association Functional Classification; PCI, percutaneous coronary intervention; TA, transapical; TAVI, transcatheter aortic valve implantation; and TF, transfemoral.

a Defined as creatinine >200 μmol/L or dialysis for renal failure.

### Relative Survival

**Figure 1:**
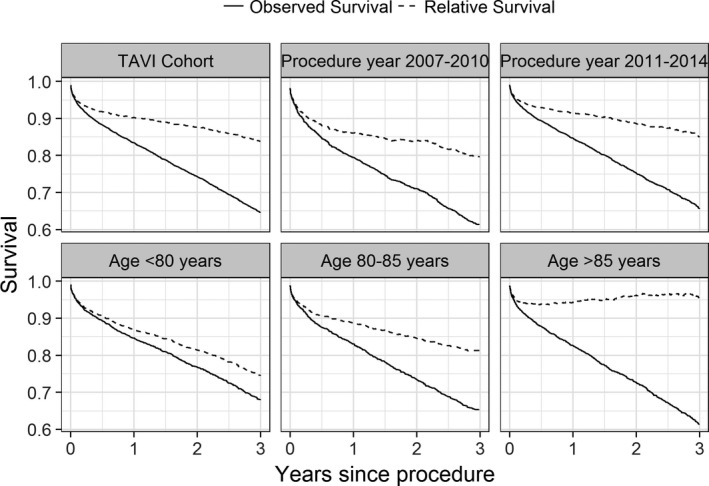
Kaplan‐Meier curves of observed and relative survival (RS). An RS of 1 implies the observed survival is the same as the expected survival in the matched general population. TAVI indicates transcatheter aortic valve implantation.

**Figure 2:**
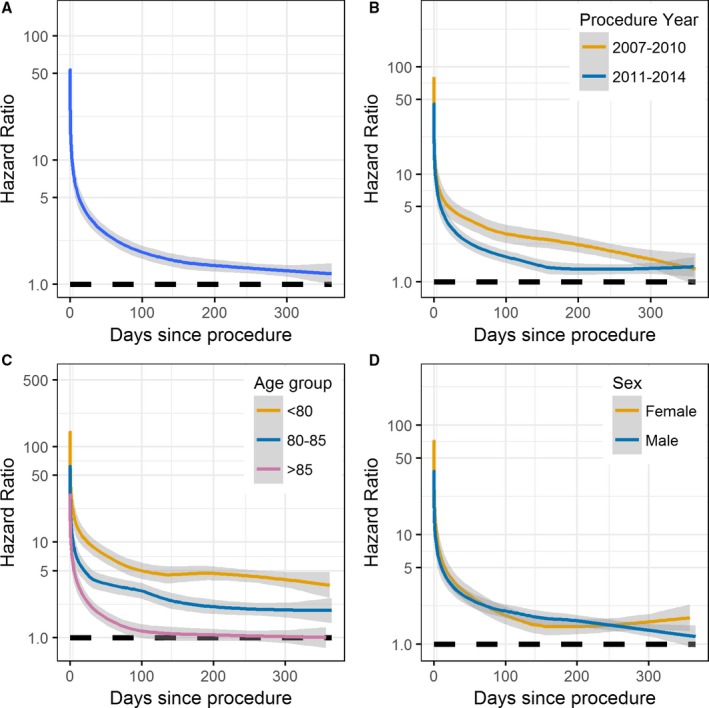
Daily hazard ratios (HRs) for the whole transcatheter aortic valve implantation cohort (A) and across subgroups of procedure year (B), age groups (C), and sex (D). Estimated daily HRs were calculated as the observed hazard of mortality divided by the expected hazard from the matched general population. The lines are the smoother through each of the estimated daily HRs, with 95% confidence intervals shaded.

**Figure 3:**
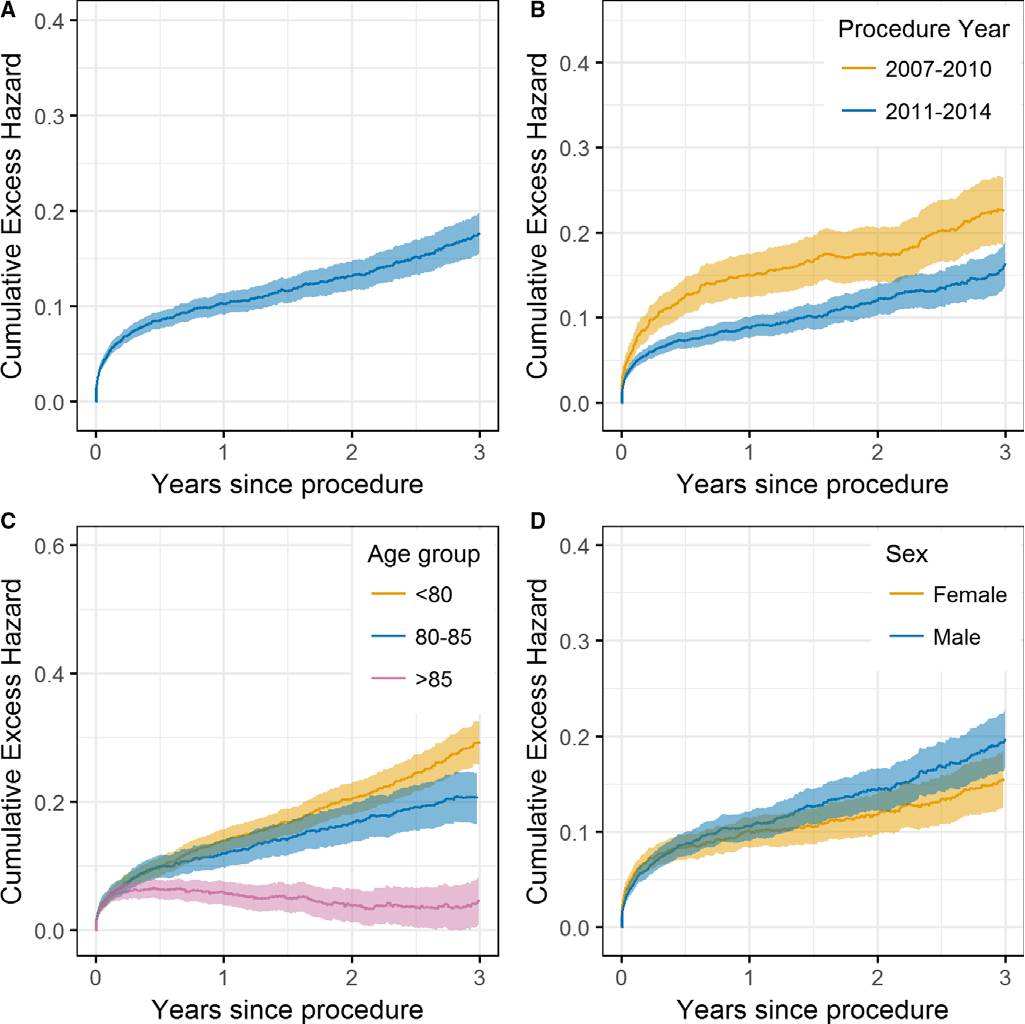
Cumulative excess hazard curves for the whole transcatheter aortic valve implantation (TAVI) cohort (A) and across subgroups of procedure year (B), age groups (C), and sex (D). An increasing (decreasing) cumulative excess hazard indicates worse (better) mortality hazard in the TAVI population compared with that expected of the general population. A cumulative excess hazard of 0 implies that the observed survival is the same as the expected survival from the general population.

In the TAVI cohort, all‐cause 30‐day, 1‐year, and 3‐year observed survival estimates were 94.7%, 83.4%, and 64.5%, respectively, compared with corresponding RS rates of 95.4%, 90.2%, and 83.8% (Figure 1). Hence, immediately after the procedure, the hazard of mortality in the TAVI cohort was ≈50 times higher than that of the matched general population (Figure 2A). However, the daily HR decreased rapidly, and by 1 year, the observed mortality rate within the TAVI cohort was only 1.26 times higher than that of the matched general population (HR, 1.26; 95% confidence interval, 1.05–1.46). After 1 year, the cumulative excess hazard curve increased at an approximately constant rate; thus, the TAVI cohort had marginally higher mortality hazards compared with the general population up to 3 years after the procedure (Figure 3A).

RS was higher in 2011 to 2014 than for procedures conducted in 2007 to 2010 (Figure 1). In particular, the increase in mortality hazard over that in the general population immediately after the procedure was greater in 2007 to 2010 than in 2011 to 2014, with the daily HRs decaying quicker for the 2011 to 2014 group (Figure 2B). By 1 year, the daily HR for both procedural year groups was similar. Consequently, after 1 year, the cumulative excess hazard curves increased at a similar rate, meaning that the initial elevation in mortality risk for earlier procedures relative to the general population persisted throughout follow‐up (Figure 3B).

By 100 days after TAVI, the observed mortality rates within the >85 years age group were the same as the expected mortality rates within the >85 years matched general population (HR, 1; Figure 2C). Such a finding was not observed in the <80 or the 80 to 85 years age groups, with the observed mortality hazard in these groups being higher than that expected from the matched general populations throughout follow‐up (Figure 3C). In contrast, for the >85 years age group, the cumulative excess hazard curve plateaued by 1 year (because the HR was 1); and by 3 years, the cumulative excess hazard curve had decreased towards 0 (Figure 3C). Therefore, after surviving the initial high risk of the procedure, the observed survival rate in the >85 years age group was approximately similar to that in the >85 years general population by 3 years (RS≈1; Figure 1).

**Figure 4:**
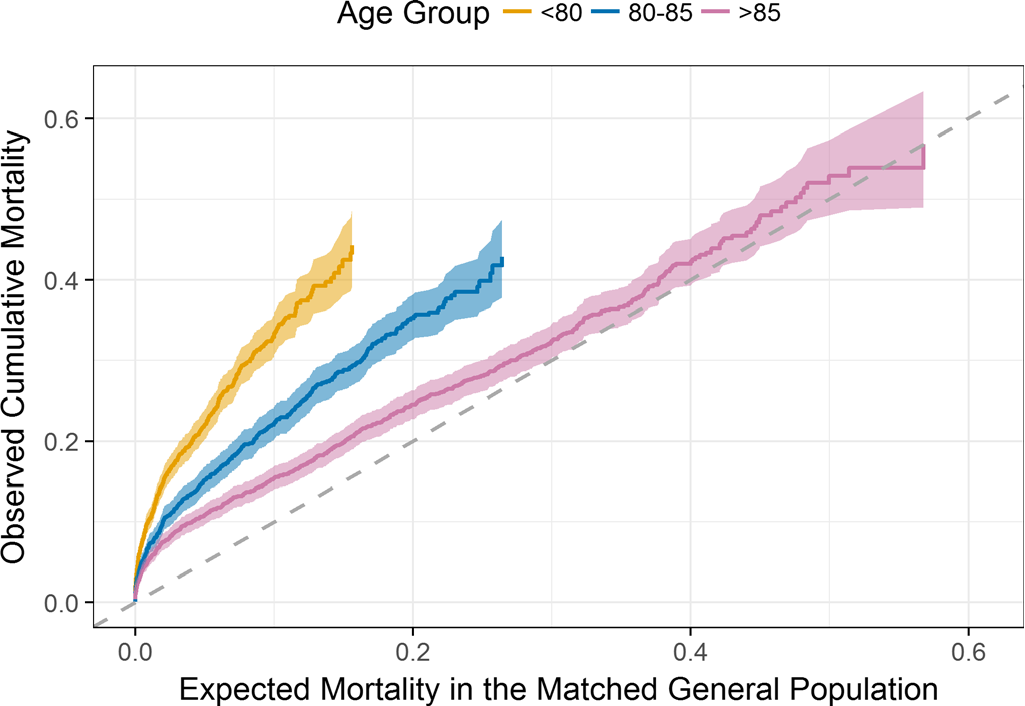
Cumulative mortality curves on the transformed time scale per age category. The horizontal axis represents the mortality rate expected in each representative population, whereas the vertical axis shows the observed mortality on this transformed time scale. The dashed line represents the null hypothesis that the matched background mortality rates apply.

Figure 4 shows the survival curves per age group after transforming each patient's follow‐up time (in days) to indicate population‐expected mortality. For example, when the expected mortality rate in each representative population was 20%, the observed cumulative mortality rate was ≈25% in the >85 years age group and 36% in the 80 to 85 years age group. Hence, Figure 4 indicates that, by the end of follow‐up, the mortality rates observed within the >85 years age group approximated those expected from the general population (dashed line), with RS significantly improved with increasing age (log‐rank test, *P*<0.001).

### Modelling RS

**Figure 5:**
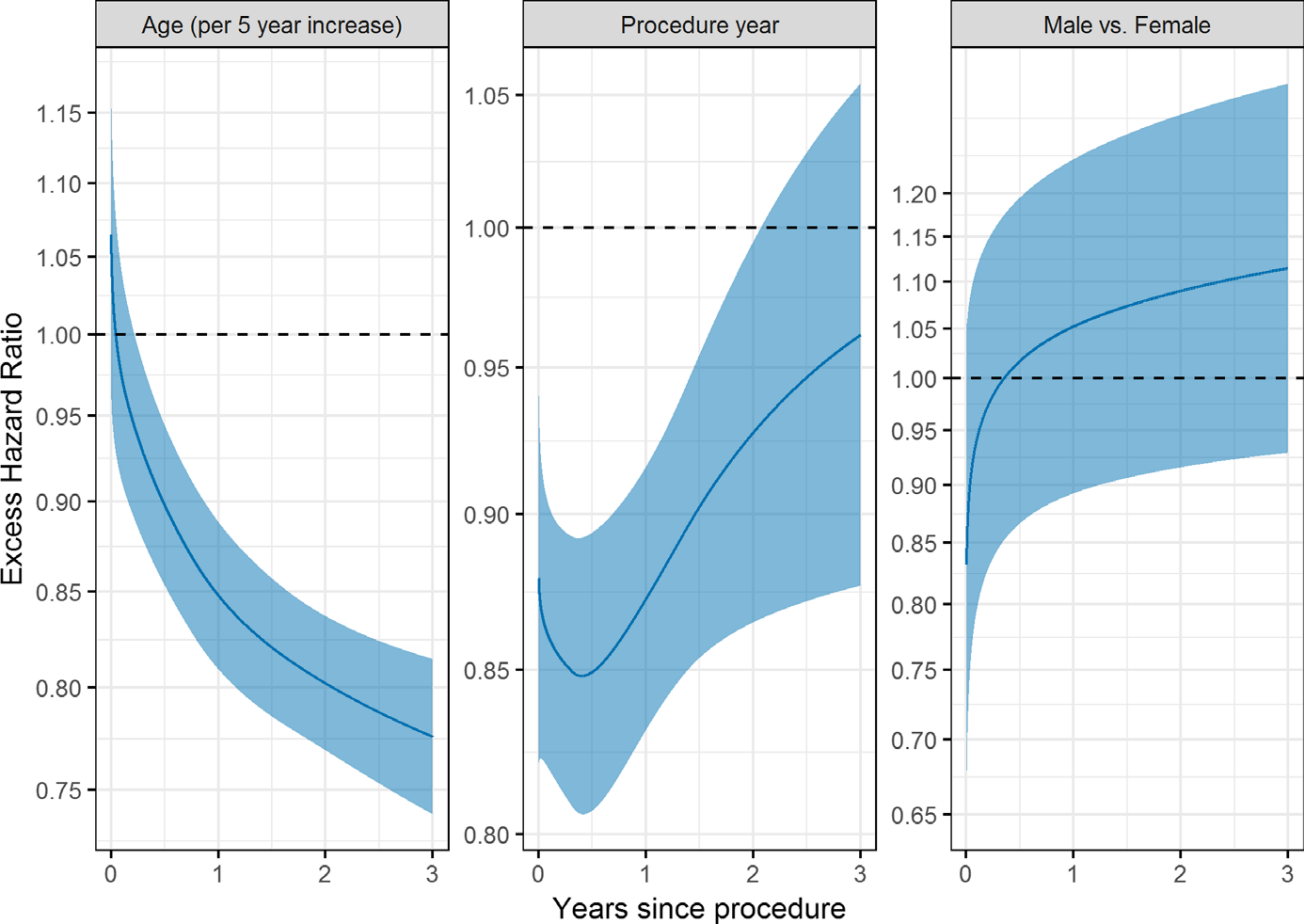
Time‐dependent excess hazard ratios (HRs) from the flexible parametric relative survival model. An excess HR of 1 means that the excess hazard (over that in the general population) was the same between groups.

**Figure 6:**
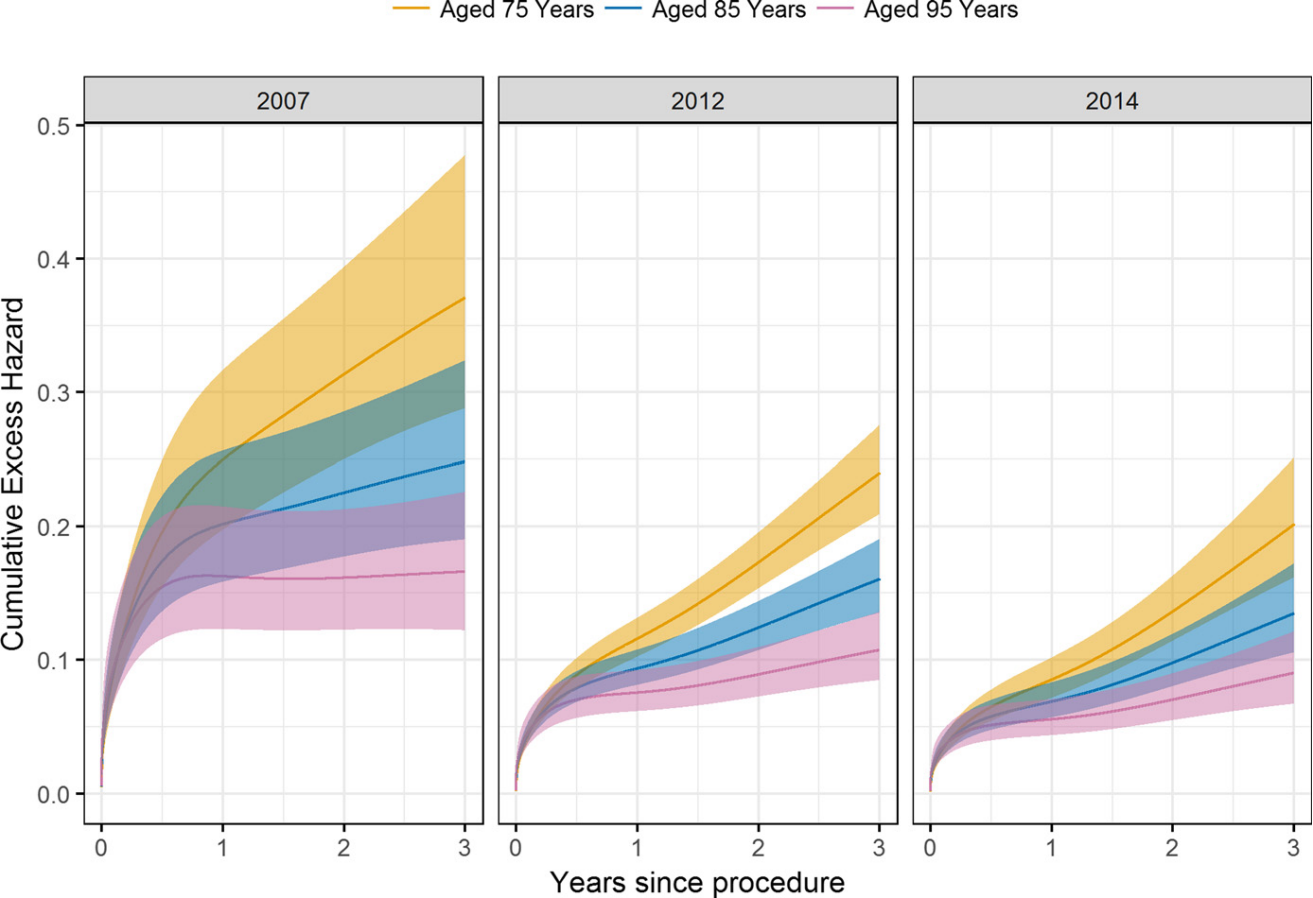
Predicted cumulative excess hazard curves for patients aged 75, 85, and 95 years, across increasing procedure year, obtained from the flexible parametric relative survival model. The 95% confidence intervals are indicated by the shaded areas.

Figure 5 gives the time‐dependent excess HRs for age (per 5‐year increase), procedure year (number of years from 2007), and sex from the flexible parametric RS model. Increasing age was associated with significantly lower excess hazards after ≈3 months post procedure. For example, at the 2‐year follow‐up, the excess HR for age (per 5‐year increase) was 0.80 (95% confidence interval, 0.77–0.84) (Figure 5). Thus, on average, a given patient was experiencing 20% lower excess mortality over the general population compared with a similar patient aged 5 years younger. Moreover, this model highlighted significant temporal improvements in RS for midterm follow‐up, with later procedure years having excess hazards significantly <1, until 2 years after the procedure. The joint effects of age and procedure year on cumulative excess hazard are illustrated in Figure 6. RS was similar between male and female patients throughout follow‐up (Figure 5), supporting the findings shown in Figure 2D and Figure 3D.

## Discussion

This analysis of the UK TAVI registry aimed to compare the observed survival against that expected from a matched general population. We found that, although the observed mortality hazard was high relative to that in the general population immediately after TAVI, the HR decayed rapidly within the first year of follow‐up. After 1 year, RS was significantly higher than all‐cause survival, indicating that a large proportion of the long‐term mortality can be accounted for by the underlying mortality risks within the general population. By 1 year, most elderly patients were experiencing mortality hazards comparable to the general population and by 3 years, the excess hazard had reduced sufficiently for the observed survival to return to population‐expected survival rates. Finally, our analysis shows that there were significant improvements in RS for those who underwent TAVI in 2011 to 2014 compared with 2007 to 2010.

The 1‐year all‐cause survival rates reported in this study compare with those from other national registries,^7^, ^9^, ^25^, ^26^, ^27^ but these do not account for competing causes of death. Without cause of death information, the expected mortality risks derived from the life tables are a suitable proxy for competing causes of death, given the low prevalence of TAVI procedures undertaken across the general population. We found that 1‐ and 3‐year RS estimates of 90.2% and 83.8%, respectively, were significantly higher than the corresponding all‐cause survival rates of 83.4% and 64.5%. Thus, the initially high excess mortality, induced by the index AS and associated TAVI, decreased significantly within the first year. This supports previous work using cause of death information, which demonstrated that, although most short‐term mortalities were cardiovascular and procedure related, beyond 24 months, noncardiovascular causes became the leading cause of death.^25^ Moreover, the RS rates reported in this study approximately compare with the cardiovascular causes of death reported in the high‐ and intermediate‐risk PARTNER (Placement of Aortic Transcatheter Valves) randomized trials.^3^, ^5^ For example, the intermediate‐risk PARTNER trial reported a 1‐year cardiovascular death rate of 7.1% and the high‐risk trial reported a corresponding rate of 14.3%; these values compare with our reported 1‐year RS mortality rate of 9.8%. Arguably, RS estimates are more useful to healthcare users and providers than all‐cause analyses, particularly given the decline in cardiovascular causes of death.^26^ By examining RS, this analysis suggests that the mortality risks observed after TAVI return to those expected within a general population, thereby supporting trial data showing the effectiveness of the procedure.^2^, ^3^, ^5^

High rates of RS were particularly evident in most elderly patients. Older age has previously been a significant predictor of 1‐year mortality after multivariable adjustment,^9^ with nonagenarians particularly being associated with increased risk.^27^ However, by adjusting for mortality risk within the underlying population, the present work demonstrates that elderly patients with TAVI had better survival than younger patients did, relative to their matched general populations. Survival in the oldest patients was equivalent to that of a matched general population by 3 years, which supports the work of Arsalan et al.^27^ These researchers demonstrated that, despite the increased mortality risks of nonagenarians, the relative difference between the observed rates and an age‐matched general population were less than for those aged <90 years. Although one needs to consider survival bias, the current work suggests that a large proportion of long‐term deaths in elderly patients undergoing TAVI are unrelated to the procedure or the index AS. This has important clinical implications from a resource use perspective, but further studies on national registries that exploit administrative cause of death data are required.

Nevertheless, because TAVI is predominantly undertaken in patients who are considered high surgical risk, a patient aged <80 years who undergoes TAVI is likely to have a range of comorbidities that will influence subsequent survival. Hence, the RS estimates for young patients undergoing TAVI might be biased because we could not adjust for a correspondingly comorbid young general population. Similarly, after surviving the initial high‐risk period after TAVI, elderly patients are logically more robust than their counterparts in the general population. Hence, given octogenarians survive the initial high risks, it is unsurprising that they compare with the matched general population. Cardiac teams will select patients cautiously in the eldest age group, and this could lead to selection bias. Paradoxically, the older patients undergoing TAVI had fewer baseline risk factors than the younger patients did, likely because of careful patient selection (Table 1). The current analysis suggests that given such careful selection practices, elderly patients should continue to be considered for TAVI, given that they appear to tolerate the risks of the procedure well and their mortality risks “return to baseline” within 1 year. Recent trial data highlight a potential expansion of TAVI into intermediate‐risk patients,^5^ and there is growing interest in identifying cases where TAVI will be futile.^28^ In terms of AS‐related mortality, the current study highlights that elderly patients are still viable and appropriate candidates. Arguably, one should consider improvements in quality of life and readmissions when debating if TAVI should be undertaken in the elderly patients, particularly for cost‐effectiveness estimation. Such data were unavailable in the current analysis. Consequently, although the mortality risk in elderly patients returned to that of the matched general population, we were unable to investigate if there were corresponding improvements in patient quality of life.

Finally, there have been rapid temporal developments of TAVI procedure technology and practice. This study found that the excess mortality, over that in the general population, immediately after TAVI was lower for patients who underwent the procedure between 2011 and 2014. The reasons behind the faster decrease in excess hazards for the 2011 to 2014 procedure years are unclear from the current work, but likely reflect the changes in patient selection/risk and advances in procedural techniques/valves. Mortality rates after diagnosis of AS are decreasing with time,^29^ with the current work suggesting similar temporal improvements after TAVI after accounting for competing causes of death.

### Limitations

Several limitations need to be considered when interpreting the results of this study. One of the main limitations is that associations cannot be interpreted as casual but rather highlight those needing explanation. As discussed above, a limitation of the method is that there is selection bias in the entire TAVI cohort, which might have led to a false‐negative result in the younger patients. An assessment of all patients with AS (treated surgically, by TAVI, or conservatively) might negate such selection bias, but these data were unavailable; we recommend further work in this area. Similarly, it is impossible to determine what drives excess mortality because the RS could not be decomposed into that from AS, that from TAVI, and that from comorbidities that are correlated with AS. Indeed, the Office for National Statistics life tables do not stratify by comorbidities observed in the TAVI registry, which could influence the conclusions about the age and year subgroups. In addition, the population life tables lag the census information in the UK TAVI registry, with this analysis assuming the life tables in 2015 were the same as those in 2014. Although this could lead to bias in the calculation of expected mortality rates for 2015, the amount of variation in the life tables between years for any given age, sex, and country stratum was minimal. Finally, we were only able to investigate mortality as an end point, without data on quality of life that are increasingly being used to identify TAVI effectiveness in elderly patients.

## Conclusions

In conclusion, this study demonstrates good long‐term RS in patients undergoing TAVI procedures in the United Kingdom. After surviving the initially high‐risk period after TAVI, survival in elderly patients returned to that expected within the general population by 3 years.

## Sources of Funding

This work was funded by the Medical Research Council through the Health e‐Research Centre, University of Manchester (MR/K006665/1), and a grant through the North Staffordshire Heart Committee.

## Disclosures

None.
